# An Ounce of Prevention is a Ton of Work: Mass Antibiotic Prophylaxis for Anthrax, New York City, 2001

**DOI:** 10.3201/eid0906.030118

**Published:** 2003-06

**Authors:** Susan Blank, Linda C. Moskin, Jane R. Zucker

**Affiliations:** * Centers for Disease Control and Prevention, Atlanta, Georgia, USA; † New York City Department of Health and Mental Hygiene, New York, New York, USA

**Keywords:** anthrax prophylaxis, bioterrorism, public health response, policy review

## Abstract

Protocols for mass antibiotic prophylaxis against anthrax were under development in New York City beginning in early 1999. This groundwork allowed the city’s Department of Health to rapidly respond in 2001 to six situations in which cases were identified or anthrax spores were found. The key aspects of planning and lessons learned from each of these mass prophylaxis operations are reviewed. Antibiotic distribution was facilitated by limiting medical histories to issues relevant to prescribing prophylactic antibiotic therapy, formatting medical records to facilitate rapid decision making, and separating each component activity into discrete work stations. Successful implementation of mass prophylaxis operations was characterized by clarity of mission and eligibility criteria, well-defined lines of authority and responsibilities, effective communication, collaboration among city agencies (including law enforcement), and coordination of staffing and supplies. This model can be adapted for future planning needs including possible attacks with other bioterrorism agents, such as smallpox.

As part of national bioterrorism preparedness efforts, New York City began actively developing protocols for the distribution of mass antibiotic prophylaxis against anthrax in early 1999. These efforts were led by the Mayor’s Office of Emergency Management, in close collaboration with the New York City Department of Health (DOH). The goal of the plan was to have the ability to provide mass antibiotic prophylaxis to 8 million New Yorkers over a 48-hour period—in the worst-case scenario of a large-scale bioterrorism attack—without impinging upon the capacity of local medical facilities to respond to the needs of persons affected by the biological agent. Here, we highlight aspects of New York City’s emergency planning, the circumstances of the six actual implementations in the city in 2001, and the lessons we learned.

## Planning

New York City’s government agencies, including DOH, are part of an incident command structure that reports to the Mayor’s Office of Emergency Management during public emergencies (*1*). In 1999, this department established an internal incident command structure, composed of the following components: clinical response, sheltering, surveillance, environmental health, laboratory, communications, management information systems, and physical plant operations components. “Round-the-clock” coverage was adopted at all agency levels. These teams are operated by persons from a variety of the city’s DOH programs.

## Chronology of Events

On October 9, 2001, New York City’s DOH was notified of a possible case of cutaneous anthrax in a female staff member of a nightly news team at a large media company (Table 1). On October 10, the department’s incident command system was put into effect, and team leaders were informed of the situation. From then until October 12, 2001, when the diagnosis was confirmed, DOH finalized an antibiotic distribution plan, including development of a medical charting system, standing orders for dispensing antibiotics, training curricula for staff, and reproduction of antibiotic fact sheets (in English and Spanish). Clinical materials were reviewed by the department’s general counsel, and scripts were developed for information hotlines. DOH staff were identified and reassigned to this effort.

**Table 1:** Chronological summary of six anthrax events requiring PODs^a^

Event	Location	No. of eligible persons registered	Total hours of operation	Briefing format for eligible persons oral/written	Antibiotics	Nasal swabs taken
1	Media 1	1,322	42	No/yes	Yes	Yes
2	Media 2	763	36	No/yes	No	Yes
3	Media 3	175	25	Yes/yes	No	Yes
4	Media 4	354	14	No/yes	No	Yes
5	USPS	7,081	67	Yes/yes	Yes	No
6	Hospital	1,923	28	No/yes	Yes	No

^a^POD, point of distribution (for antibiotics); USPS, U. S. Postal Service.

On October 12, 2001, the department began collecting nasal swabs and distributing prophylactic antibiotics to persons working at the media company who might have been exposed to a letter implicated in the index case. Included in this effort were those working on the same floor as the index patient. Initially, the exposure source was believed to be a letter postmarked September 25, 2001, and potentially exposing an estimated 200 persons. This letter was tested for *Bacillus anthracis* multiple times, however, and all tests were negative (*2*).

Within hours of the Mayor’s public announcement of this case, DOH and the Office of Emergency Management established an antibiotic distribution site (referred to as a point of distribution [POD]), at the main building that housed the media company. The space provided for prophylaxis was in the same building complex that housed the letter but did not share the ventilation systems that served the areas in the letter’s path. The layout of the space provided for the POD and its operations could not accommodate large groups of people seeking antibiotics. Moreover, the letter was a matter of a criminal and epidemiologic investigation, so law enforcement agencies needed to conduct their own interviews on site. Thus we coordinated with law enforcement personnel to minimize disruption of client flow and ensure that client medical confidentiality would not be compromised. The epidemiologic aspects of the investigation were initially incorporated into the medical record used.

Soon after distribution of antibiotics was begun, the source of anthrax was confirmed to be a letter postmarked September 18, 2001. Consequently, the time interval during which exposure may have occurred was reevaluated and the number of people possibly exposed substantially expanded.

Between Friday, October 12, and Tuesday, October 16, after approximately 42 hours of operation and an average of 55 staff persons per shift, 1,322 persons were briefed, completed epidemiologic and law enforcement interviews, underwent medical assessments, had nasal swabs taken to better define exposures, and were given a 14-day supply of antibiotics within the POD space. The average throughput time (the time from a client’s entry into the POD space to exit) was 30 minutes per client. Initially, the briefing of staff consisted of providing written materials. This system was augmented by a combination of information distributed over closed-circuit television throughout the still-operating company and by direct electronic communication from the company’s senior management. Within the first day of operations, it became apparent that both potentially exposed and unexposed persons needed emotional support and further information about the event, the risk for anthrax exposure, and the dangers of antibiotic misuse. Counselors (medical and mental health) were made available immediately outside the POD, and hotline staff were given scripts to assist them in answering concerned callers. The city’s DOH supplied each potentially exposed person with an initial 2-week course of antibiotics to provide time for public health officials to complete the investigation and develop specific criteria for persons needing to complete the balance of the 60-day prophylactic regimen.

Once the investigation was complete (October 20, 2001), DOH narrowed the criteria for antibiotic prophylaxis to those met by the 12 persons who directly handled the contaminated letter and recommended that all others discontinue antibiotics. This general information was communicated by the employers to all antibiotic recipients and by letters mailed from DOH to affected persons. We also directly contacted all 12 persons who needed to continue prophylaxis. Ultimately, 60-day inhalational anthrax prophylactic regimens were provided to 11 persons (6 working in the building and 5 involved in the recovery of the tainted letter) by means of the on-site employee health unit. One person refused prophylaxis. We later assisted the Centers for Disease Control and Prevention in evaluating adherence and adverse drug effects among those receiving 60-day regimens.

Four cutaneous anthrax cases were subsequently identified in New York City; these cases occurred at three other media outlets (one case each at two locations and two cases at the third) (*3*). All these cases were believed to be associated with contaminated mail. No inhalational anthrax cases were associated with the media outlets. These three PODs served persons potentially in direct contact with the suspect letters. POD activities, however, were restricted to registration, provision of printed information, epidemiologic interviews, and obtaining of a very limited number of nasal swabs within the POD space. Subsequently, the decision to provide antibiotics was based on confirmed exposure, as determined by the epidemiologic investigation. Antibiotics were dispensed on an individual basis, as was monitoring for adherence and adverse events. Epidemiologic and law enforcement interviews and large informational sessions for all staff were held separately, in separate facilities, and at different times from those for the POD. Counseling was available immediately after the information sessions or thereafter through the DOH anthrax hotline.

The fifth POD was conducted in New York City by the U.S. Public Health Service (USPHS). This site’s purpose was to provide an initial 10-day course of antibiotics to prevent inhalational anthrax in ~7,000 postal employees who worked at facilities that processed the anthrax-containing letters sent to the above referenced media outlets (events 1–4). Although no anthrax cases had been reported among the city’s postal workers, inhalational anthrax cases had occurred in postal workers in New Jersey and in the Washington, D.C., area (*4*). Anthrax spores were subsequently found in one of New York City’s postal facilities. Both labor and management at affected facilities requested prophylaxis for inhalational anthrax. As these were federal facilities and federal employees, prophylaxis efforts remained in the jurisdiction of the federal government. The POD was conducted by USPHS in the basement of a New York City mail-processing center (*5*). U.S. Postal Service management was instrumental in securing space and identifying and scheduling staff. USPHS determined the initial operational layout, medical charting, and staff needed for this effort on the basis of its prior experiences in the Washington, D.C., area postal facilities. Additionally, written information was deemed insufficient for this setting. The increased throughput time reflects the inclusion of extensive live briefings accommodated within this POD space.

Liaisons from DOH’s clinical response team were assigned to this effort as consultants. DOH’s role was limited to increasing the efficiency of POD operations. We assisted USPHS effort by providing detailed clinician training materials, medication fact sheets, and on-site flow analyses with recommendations to improve client throughput on the basis of local POD experiences. Collaborative efforts also included the timely sharing of information with DOH for response to public inquiry, DOH assistance in establishing local medical and mental health referral patterns, and follow-up of these referrals. USPHS, in turn, accommodated visits to the operation by members of the New York City Office of Emergency Management and DOH staff for educational purposes.

Between Wednesday, October 24, and Saturday, October 27, in approximately 67 hours of operations with 65–70 staff persons per shift, this fifth POD provided 7,081 persons with a 10-day supply of antibiotics. The POD provided registration; completion of a medical screening form; detailed live briefings on risk for exposure, signs and symptoms of anthrax, and side effects of the recommended antibiotics; medical screenings; and antibiotic distribution. The average throughput time for these activities was 33 minutes per client. In addition to the medical personnel who were on site to evaluate symptoms and adjust antibiotic regimens, staff were available for mental health and other counseling issues. Epidemiologic and law enforcement interviews were conducted separately; no nasal swabs were collected because >30 days had elapsed since the suspect letters were processed. The federal agencies directly managing prophylactic efforts subsequently offered additional prophylactic antibiotics with or without the anthrax vaccine to those persons thought to have been most highly exposed to aerosolized *B. anthracis*.

On October 28, 2001, DOH was notified of a case of inhalational anthrax in a 64-year-old woman working in a hospital stockroom. The patient had no discernable association with the media companies or the postal service, although a section of the stockroom where she worked was adjacent to the hospital mailroom (*6*,*7*). While environmental samples were being collected, DOH immediately set up a POD (event 6) for hospital staff, patients, and visitors who had spent >1 hour in the hospital since October 11 and thus might have a risk for exposure to aerosolized *B. anthracis.* During the environmental investigation, the hospital was closed.

Between Monday, October 29, and Friday, November 2, over the course of 28 hours and with a staff of 53 persons per shift, 1,923 persons received prophylactic antibiotics. Epidemiologic and law enforcement interviews were targeted to include only hospital staff. Nasal swab specimens were collected from 28 persons who worked in and around the mailroom. The average POD throughput time was 6½ minutes per person. POD activities involved registration, triage, medical evaluation, dispensing antibiotics, counseling, and overall management. No informational sessions were conducted; however, written information (including DOH hotline telephone numbers) was distributed, and counseling staff were available. Nasal swabs were not routinely collected. This POD, which was situated in a hospital and focused on hospital personnel, was facilitated by close collaboration with the hospital administration, which helped coordinate prophylaxis efforts and mobilize hospital staff to assist in POD operations.

Antibiotic distribution was discontinued on November 2, when all environmental samples from the hospital tested negative for *B. anthracis.* By mail, DOH informed all persons provided with antibiotic prophylaxis to discontinue their regimens.

## Discussion

### Planning versus Reality

Prior emergency planning addressed large-scale events affecting 8 million New York City inhabitants; under those circumstances, ordinary medicolegal considerations would not apply (e.g., no provider-patient relationship invoked; no need for medical charting, nonprofessionals used for staffing). Our PODs were initiated before the extent of exposure was known and were later limited to those persons most likely to have been exposed. Clearly, the intentional release of anthrax affected far fewer than the projected worst-case scenario.

Consequently, our PODs were more “classically” modeled, i.e., they included a large staff of licensed medical professionals who obtained consents, took medical histories, collected specimens, and dispensed antibiotics. A full medical charting system was available, as were mental health and medical counseling services, at each site. These services were augmented by toll-free hotlines.

### Client Screening, Functional Units, and Flow Patterns

Clients were persons meeting eligibility criteria for receiving antibiotics and thus granted access to the POD. Ineligibles, or the “worried well,” were persons who did not meet the eligibility criteria to enter the POD or receive antibiotics within the POD; they were offered informational materials, the opportunity to speak with counselors, and access to the DOH public hotline.

The POD proper is defined as the space where patients are registered, triaged, have swab samples taken (as necessary), evaluated medically (as necessary), and provided with antibiotics. Other POD-related activities (which may or may not be part of the layout of the actual POD space) include assessing the eligibility of persons who present themselves, reassuring the worried well, briefing clients about anthrax and POD operations, collecting information for investigative purposes, transferring persons to a medical facility (when needed), counseling, managing client flow, and maintaining security.

Immediately outside the entrance to the POD, we placed a screening station, where POD staff verified eligibility and gave eligible persons writing tools, an information sheet, the epidemiologic interview form, the law enforcement interview form, and a medical record form to complete. Articulating clear eligibility criteria and obtaining verifiable lists of names of persons expected at the POD (including relevant contractors such as housekeeping and house security) helped maintain order at the front door. As these events occurred in occupational settings, management was critical in communicating public health messages to staff, identifying and scheduling staff access, and setting clinic hours to maximize the flow of the prophylactic effort; strong management resulted in organized PODs and responses.

Because bioterrorism is a criminal act, law enforcement agencies had a separate and independent purview for investigation. Performing investigative interviews first and separately from the POD proper alleviated concerns about maintaining client medical confidentiality and facilitated client flow, although this was dictated largely by the layout and physical capacity of the space allotted. Furthermore, investigative interviews involved more well-defined and smaller subsets of persons with each subsequent POD.

The client registration process also evolved with each POD. Initially, identifying data were handwritten in a logbook. This system was supplanted by the use of a single spreadsheet on a laptop, and finally, by the second day of the first POD, by several laptops with data-entry screens and wireless connections to an on-site server. These adaptations permitted rapid tracking of clients served and facilitated subsequent correspondence through the production of mailing labels. This system was upgraded and used at subsequent DOH PODs. As we quickly adopted a computerized registration process, management information system staff provided on-site technical support.

After registration, clients moved into the triage area, where an assessment was made about whether they could proceed directly to the dispensing station or needed to be medically evaluated before a final determination on prophylactic antibiotics could be made. All clients not eligible for immediate receipt of prophylactic antibiotics were triaged to the medical evaluation area. There, staff (physicians, nurses, and physician assistants) determined the appropriate prophylactic medication choice or need for further evaluation and transfer to a healthcare facility. Because a limited number of circumstances require alteration of the prophylactic regimen or of a client’s original medication regimen (~10% of all clients), we created a clinical algorithm and preprinted instruction sheets for those situations.

Antibiotics were distributed at the dispensing station, as were fact sheets explaining antibiotic use. This station was staffed by nurses, physicians, or pharmacists, as resources permitted. Having at least one pharmacist present proved useful.

Because some clients were overwhelmed by the situation and had residual questions, mental health, medical advisors, and public health educators were available at the POD entrance and near the POD exit for consultation. Also, by referring persons to the hotline and website, we limited the need for on-site counselors.

Security is an essential feature of POD operations. The New York City Police Department provided this service. Officers maintained order at the entrance and exit, so that the POD was not overwhelmed with anxious and angry persons (either those at risk or the worried well), and guarded pharmaceutical supplies.

Persons devoted solely to ensuring the smooth flow of clients into the POD, from one area (or station) to the next, and out of the POD also were essential. These flow managers, or “traffic” personnel helped minimize client-waiting times and staff idle time and improved throughput times. An area for clerical staff to manage medical charting within the POD also was necessary.

### Tailored POD Elements

Each POD was conducted differently, combining a standardized response for anthrax prophylaxis with the unique needs of each setting. Services provided within the POD space varied. The space allotted, the POD staffing available, preexisting circumstances (e.g., organizational structures, historic relations between labor and management, client characteristics), and ongoing field assessments determined the array of services offered. Similarly, POD work-shifts were defined on the basis of need, resources, and input from representatives of those affected.

### Staff and Space Needs

**Figure 1:**
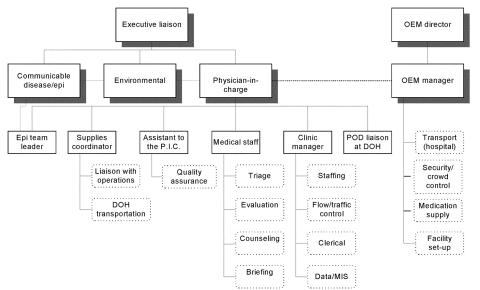
Point of distribution (POD) site organizational chart. OEM, Mayor’s Office of Emergency Management; PIC, physician-in-charge; Epi, epidemiologic; DOH, Department of Health. Dotted boxes = areas of responsibility; dotted lines = shared communications.*For operational purposes, the epidemiologic team leader reported to the PIC.

The most important element for an efficient POD process is adequate staffing to operationalize antibiotic distribution and to ensure that anticipated language needs of the clients are met. Ideally, an organizational diagram should be in place, along with a brief description of the role of each staff member and any training documents necessary. The organizational chart we found most useful is shown in Figure 1. Four critical positions are the executive liaison, physician-in-charge, supplies coordinator, and clinic manager; their primary responsibilities are outlined in Table 2. We learned that the physician-in-charge should be dedicated solely to running the POD. A second public health officer should be on-hand to convene regularly with key representatives of potentially exposed populations.

**Table 2:** Job titles and primary responsibilities recommended for PODs^a^

Job title	Primary responsibilities
Executive liaison	Reports to the incident commander
	As senior staff member in the field, coordinates both the investigation (epidemiologic and environmental) and the prophylaxis effort
	Interfaces between the public health agency and the organization representing those to receive prophylaxis
	Ensures that the physician-in-charge is informed of recent developments of the investigation, as well as other information from Department of Health command center briefings (i.e., changes in treatment recommendations, eligibility criteria, or reports of organism antibiotic susceptibilities)
Physician-in-charge	Reports directly to the executive liaison, keeping him or her appraised of progress and problems
	Is responsible for the overall POD operations, including site selection, POD set-up (including floor plan and staff training), ensuring communication among POD stations, and overseeing collection of epidemiologic and law enforcement data
	Is responsible for on-site oversight of the epidemiologic investigation, the supplies coordinator, the medical service staff (e.g., physicians, nurses, pharmacists, mental health professionals), and the clinic manager
Supplies coordinator	Ensures that all forms, supplies, and equipment are available at the POD when needed (prepared in advance, supplied to POD, and replenished as needed)^b^
	Is responsible for transportation of staff and material.
Clinic manager	Oversees nonclinical operations within the POD, such as staffing, patient flow, clerical, and MIS operations, communications, medical records retention, and quality improvement activities
	Coordinates activities with the supplies coordinator

^a^POD, point of distribution (of antibiotics); MIS, management information systems. ^b^Supplies to be provided include general supplies (medical charts, epidemiologic questionnaires, preprinted training instructions for staff at various stations, literature for patients and staff, medical charts, office supplies, white coats, and other clothing with appropriate insignia for nonclinical personnel), laboratory supplies (if needed, nasal swabs, laboratory requisitions forms, specimen bags, specimen labels, water-free hand sanitizing solution, and disposable laboratory gowns, gloves, and biohazard bags), and pharmaceutical supplies (antibiotics [in adult and pediatric dosages], a copy of the Physician’s Desk Reference, and medications fact sheets for each drug to be dispensed).

**Figure 2:**
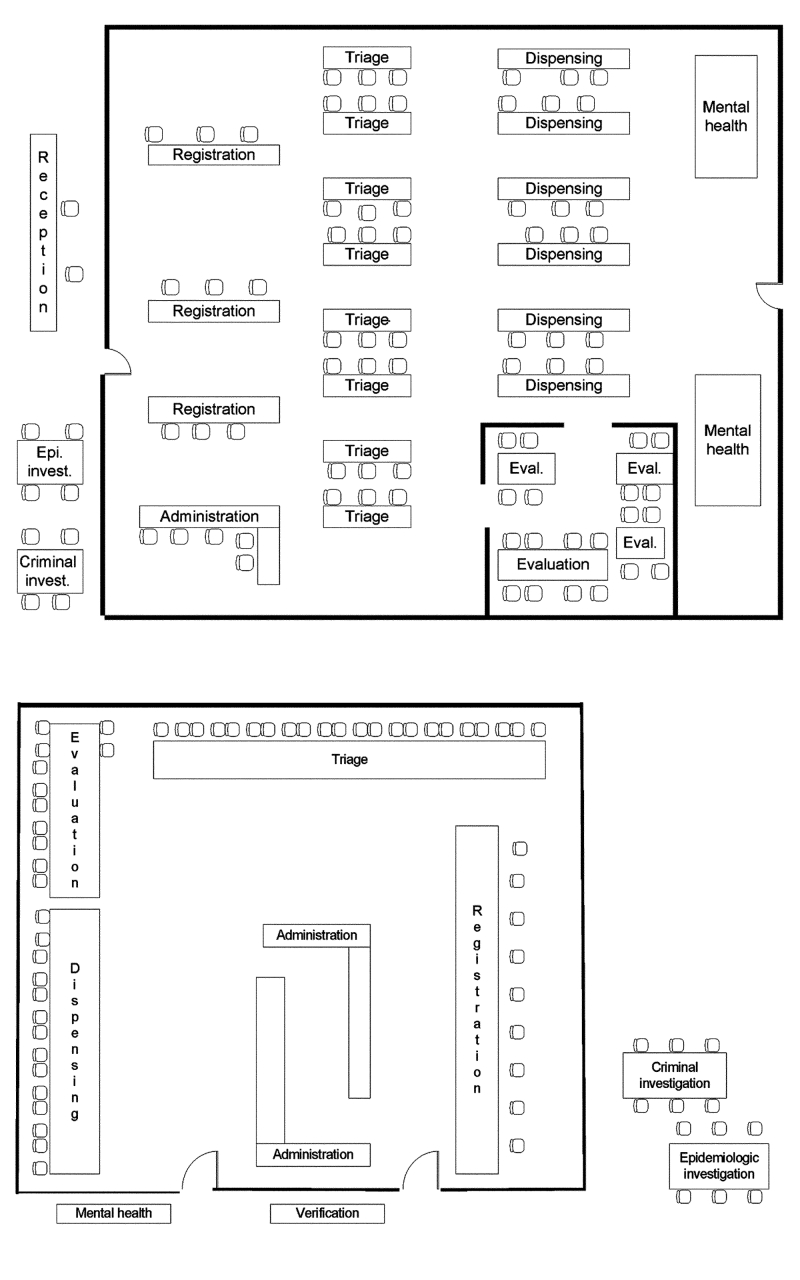
Two (A, B) point of distribution site floor plans. Epi, epidemiologic; invest, investigation; admin, administration; eval, evaluation; Disp., Dispensing; Reg, registration. B, floor plan of POD proper. The verification, epidemiology investigation, and criminal investigation sections are located before the POD proper. The mental health and briefing sections are also located outside the POD proper.

The POD site should be conveniently located for those affected but should not be located in a place that might be contaminated with *B. anthracis.* Ideally, site options should be considered well before the need for such a site arises. Selecting a space and arranging stations to promote continuous flow of clients (including the disabled and children in strollers) proved important. To distribute antibiotics to 500 to 10,000 clients over a 72-hour period, a space of at least 2,500 square feet for the POD proper was necessary. To minimize the impact of unanticipated space issues, we subsequently developed several possible floor plans, so that a quick assessment of layout could be made during subsequent site selection (Figure 2, A and B) and a predesigned floor plan could be adapted to a particular situation. Despite the urgency of the situation, allocating adequate time before opening a POD is critical to ensure that supplies have arrived and trained staff are ready to begin operations.

### Client Flow through the POD

Our PODs operated most efficiently when activities were handled at discrete workstations. As we progressed though the six events, we also realized the utility of physically separating activities for which clients may need to sit from those that did not require sitting. It was most time-efficient if clients did not sit to receive clinical services, and most space-efficient if no chairs were available for clients. Paperwork and interviews were best suited to occur outside the POD proper, since clients found it easier to fill out forms and participate in interviews while sitting. Thus space allocation should also include a space, preferably outside the POD proper, for these “seated” client activities (i.e., filling out forms and conducting interviews) to ease the difficulties of moving people through the POD. Persons able to perform briefings and translators (including those skilled in sign language) should be available in this area to assist with questions.

### Streamlined Medical Chart

The medical chart was revised between events. Initially, we obtained very structured medical histories and collected nasal swabs from all clients, creating tremendous delays. With subsequent PODs, we redesigned our medical chart to be a one-page (two-sided), self-administered questionnaire, limited to information relevant to the rapid distribution of antibiotics. The chart included personal contact information (e.g., address, telephone numbers, and identification of emergency contacts), a signed consent form for testing and treatment, brief medical history (presence or absence of current anthrax symptoms, relevant drug allergies, use of specific medications known to interact with doxycycline or ciprofloxacin, pregnancy status), as well as a place to document nasal swab collection, the dispensing and receipt (or refusal) of antibiotics, and antibiotic lot numbers. A separate medical record was created for pediatric clients and followed the same general formatting. Also, as the utility of nasal swabs became better understood, DOH progressively restricted the epidemiologic criteria for obtaining them, relieving an important system bottleneck at triage.

### Short Briefings

If necessary, live briefings need to be short and should include multiple briefing stations with good sound systems. Staggered briefings (i.e., 7–10 minutes in length, beginning every 5 minutes) helped distribute client flow. Including information on antibiotic dosage and side effects in these briefings was useful. Persons able to perform briefings and translators (including for sign language) should be available in this area to assist with questions. Clients may be provided with a written information sheet in lieu of a briefing, a step that improves client flow; a counselor can be available to handle further questions.

### Communication

Careful attention to communication at a variety of levels is critical, including from the incident command center to the POD and from the health department to the public and to community medical providers. Also important was the flow of information from public health officials to representatives of the community receiving prophylaxis, and to the community itself. Without such attention, centrally made decisions might not be communicated to POD staff, resulting in mistaken expectations.

Cell phones and two-way radios were important means by which to communicate. Electronic mail was not available for POD staff. Materials initially developed required continuous updating of facts, whether or not new information was available (e.g., “There are no new cases of anthrax as of today.”). These materials needed to be appropriate for public use. Materials were used at POD sites, for DOH hotline scripts, and on the DOH website. Information was also disseminated by means of press releases and press conferences.

The format for communicating with POD clients—including printed materials, live briefings, or both—was decided jointly by management and public health officials on the basis of resources, the extent and severity of actual cases, and knowledge level of the clientele. The medical community was kept abreast of recent developments through multiple broadcast faxes, emails, and website updates from DOH and by quickly establishing a DOH physician hotline staffed by medical professionals. In summary, DOH established three separate hotlines, one each for physicians, those clients directly affected by POD operations, and the general public.

### Preplanning

Events that require a POD (i.e., intentional dissemination of virulent organisms) are stressful for all involved. In a public health emergency, little time exists between the decision to open a POD and initiation of operations. Planning can help alleviate the need to make decisions under pressure and can ensure quality of effort. The interval before opening the POD can then be used for truly last-minute preparations: mobilizing and orienting personnel, finalizing briefing sheets, and selecting a POD location and layout.

Training should also begin well before an emergency actually occurs so that staff assigned to assist with POD operations are familiar with the process, forms, and data-entry screens and have a personal emergency plan in place (e.g., child and pet care, transportation) to accommodate an altered work schedule. Having each staff person’s tasks be limited enough to be “digestible” in a short orientation session at the time of POD operations is also helpful. The local health department can prepare for mass prophylaxis efforts by developing a standing set of employee rosters for round-the-clock coverage in 12-hour shifts, with approximately 50–55 persons per shift. This schedule enables antibiotic prophylaxis to be provided to up to 10,000 persons in 72 hours.

### Advance Resource Building

A major difficulty in staffing a POD with health department staff, especially in small health departments, is that these staff are removed from their regular duties. One approach to minimize the effect on single programs is to compose teams from a variety of programs. Another strategy is to use staff from preexisting program groupings, with existing work relationships.

Most health departments are not poised to handle single large POD efforts (>10,000 persons) or even multiple concurrent ones for <10,000 persons. To preserve the integrity of public health functions during large or concurrent POD mobilizations, partnerships are necessary to mobilize qualified personnel from a variety of resources in and around the affected community. Thus, health departments need to have established relationships with other organizations (e.g., Visiting Nurse Service, American Red Cross) that can assist if needed. Any mobilization across agencies will be facilitated by prior communication and coordination on issues such as deputization,^2^ licensure, medico-legal responsibility, and payment of wages.

### Allocation of Resources

Relationships arose during the POD events that made important resources available. Our prophylaxis efforts took place in occupational settings, primarily for employees at these settings. Management and labor representatives were important assets for facilitating POD operations.

DOH tried to limit antibiotic distribution to those who needed them; the department used the opportunity to educate the public on the hazards of inappropriate antibiotic use. Our role was to ensure access to antibiotics, educate POD clients of the need to complete the prescribed regimen once started, and ensure that the health department maintained critical public health functions. The concern with anthrax is primary, not secondary, spread, and as such, precious public health resources should not be used to ensure adherence on a case-by-case basis.

## Conclusion

A successful POD is characterized by clarity (clear mission and eligibility criteria, clear lines of authority, clearly defined responsibilities, clear antibiotic recommendations); communication (between the DOH incident command on site at the POD and organizations representing those receiving prophylaxis); collaboration (with other agencies that may be called upon to assist in delivery of prophylaxis and law enforcement agencies needing to gather information about the crime scene); coordination of staffing and supplies; and prudent choice of POD site. Future planning should include scenarios that address alternative prophylactic modalities (e.g., immunization, especially for smallpox), on-site infection control needs (such as use of masks or isolation for symptomatic persons), and automated management information systems for more efficient operations.

## Footnotes

2 Deputization formally gives a volunteer responsibilities and privileges during the temporary assignment as an agent of DOH. Responsibilities include following DOH rules on confidentiality, handling medical records, making decisions on DOH’s behalf, and stewarding resources (especially medications and equipment) according to DOH protocol. DOH will in turn offer some protections (e.g., proper equipment, malpractice coverage, worker’s compensation coverage).
